# Reversible 90-Degree Rotation of Fe Magnetic Moment Using Hydrogen

**DOI:** 10.1038/s41598-018-21712-3

**Published:** 2018-02-19

**Authors:** Chuan-Che Hsu, Po-Chun Chang, Yi-Hua Chen, Chak-Ming Liu, Chun-Te Wu, Hung-Wei Yen, Wen-Chin Lin

**Affiliations:** 1 Department of Physics, National Taiwan Normal University, Taipei, 11677 Taiwan; 2 Department of Materials Science and Engineering, National Taiwan University, Taipei, 10617 Taiwan

## Abstract

[Pd/Fe]_2_ multilayers were deposited on a flat MgO(001) to study the effect of hydrogen on magnetic interlayer coupling. Complex magnetic hysteresis behavior, including single, double, and triple loops, were measured as a function of the azimuthal angle in a longitudinal and transverse direction. With a combination of a 2-fold magnetic anisotropy energy (MAE) in the bottom-Fe and a 4-fold MAE in the top-Fe, the complex magnetic hysteresis behavior could be clearly explained. Two well-split hysteresis loops with almost zero Kerr remanence were measured by choosing a suitable Pd thickness and applying the magnetic field perpendicular to the easy axis of the bottom-Fe. The split double loops originated from the 90°-rotation of the top-Fe moment. On exposure to a hydrogen gas atmosphere, the separation of the two minor loops increased, indicating that Pd-hydride formation enhanced the ferromagnetic coupling between the two Fe layers. Based on these observations, we proposed that, by applying a suitable constant magnetic field, the top-Fe moment could undergo reversible 90°-rotation following hydrogen exposure. The results suggest that the Pd space layer used for mediating the magnetic interlayer coupling is sensitive to hydrogen, and therefore, the multilayer system can function as a giant magnetoresistance-type sensor suitable for hydrogen gas.

## Introduction

Pd has long been used as a high-efficient catalyst for the dissociation of hydrogen molecules into individual atoms^1–4^. With Pd present on the surface, hydrogen molecules in a gaseous state can be split and absorbed into the material volume, forming stable Pd-H hydride; simultaneously, possible lattice expansion and electron charge transference have also been observed^5–8^. The effects of Pd-assisted hydrogenation on magnetic properties have been reported in Pd/Fe, Co, Ni bilayers, [Pd/Co]_*n*_ multilayers, and Co-Pd alloys^9–14^. Furthermore, hydrogen absorption and desorption can induce reversible changes in magnetic coercivity, remanence/saturation ratio, magnetic moment, magnetic anisotropy energy, and microscopic domain wall motion^15–18^. These observations demonstrate the potential for using spintronic devices as hydrogen gas sensors^6,19–21^. Magnetic interlayer exchange coupling, also known as Ruderman-Kittel-Kasuya-Yosida (RKKY) coupling, has been widely studied through the application of giant magnetoresistance (GMR) in magnetic storage and magnetic field sensors^22,23^. RKKY coupling has been studied extensively by employing various noble metals (such as Au and Ag) or transition metals (such as V, Cr, and Cu) as the intermediate nonmagnetic layer^24^. Thus the hydrogen effect on RKKY coupling behavior in systems using hydrogen-dissociation catalyst as mediate layer was of great potential for applications. For instance, Hjörvarsson *et al*. reported that the magnetic ordering of the Fe layers in Fe/V(001) superlattices switched from parallel to antiparallel and from antiparallel to parallel when hydrogen was introduced to the V layers^25–27^. The major reason for interlayer coupling transitions, such as these, was not the hydrogen-induced lattice expansion, but the distortion of the Fermi surface in the V layers^25^. Klose *et al*. also reported a continuous and reversible change of magnetic coupling in an Fe/Nb multilayer induced by hydrogen charging. The authors attributed the change of coupling to hydrogen uptake, which causes changes in the effective Fermi wave vector in Nb^28^.

**Figure 1:**
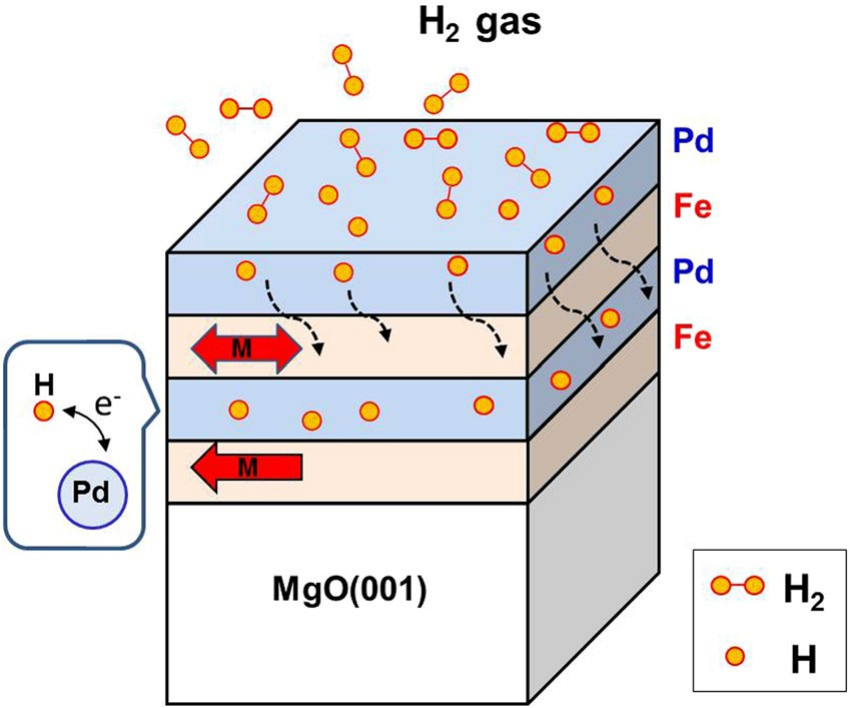
Schematic illustration of the effect of hydrogenation on the Pd covered Fe/Pd/Fe trilayers on Mg(001) substrate. Hydrogen molecules dissociated on the top Pd layer and diffused into the underlayers. The electronic structure of the Pd-mediate layer, and the interlayer magnetic ordering between the two Fe layers, is expected to be modulated through Pd-hydride formation.

Pd-related materials have been thoroughly studied and suggested as candidates for hydrogen sensors and hydrogen storage; however, very few studies have investigated Pd-mediated RKKY interlayer exchange coupling and the effect of hydrogen on the Pd-mediated interlayer coupling thus far. Furthermore, in a series of papers on the magnetic properties of ultrathin Fe(001)/Pd(001)/Fe(001)/Ag(001) structures, Celinski *et al*. reported that Pd was ferromagnetic for a thickness of up to four monolayers (MLs); correspondingly, the Fe/Pd/Fe trilayer was ferromagnetically coupled. At more than 5 MLs, the Pd interlayer was no longer ferromagnetic, and when the thickness was of 13–16 MLs, it displayed weak long-range antiferromagnetic coupling^24,29–31^. Double split hysteresis behavior was observed in the 5.7-ML Fe/14-ML Pd/5-ML Fe trilayer, indicating the presence of antiferromagnetic interlayer coupling. However, Childress studied the magnetic properties of epitaxial Fe(001)/Pd superlattices on MgO(001) and concluded that no evidence for antiferromagnetic coupling between Fe layers through Pd interlayers could be found in Pd with a thickness of 10–50 Å^32^. In the present study, as illustrated in Fig. 1, we prepared Pd/Fe/Pd/Fe multilayers on MgO(001). When the sample was exposed to H_2_ pressure, hydrogen molecules dissociated on the top Pd layer and diffused into the underlayers. The electronic structure of the Pd-mediate layer, and the interlayer magnetic ordering between the two Fe layers, is expected to be modulated through Pd-hydride formation. we examined the magnetic behavior of [Pd/Fe]_2_ multilayers and the detailed mechanism of magnetization switching was discussed.

## Experimental Result and Discussion

### Growth and crystalline structure of Pd/Fe/MgO(001)

**Figure 2:**
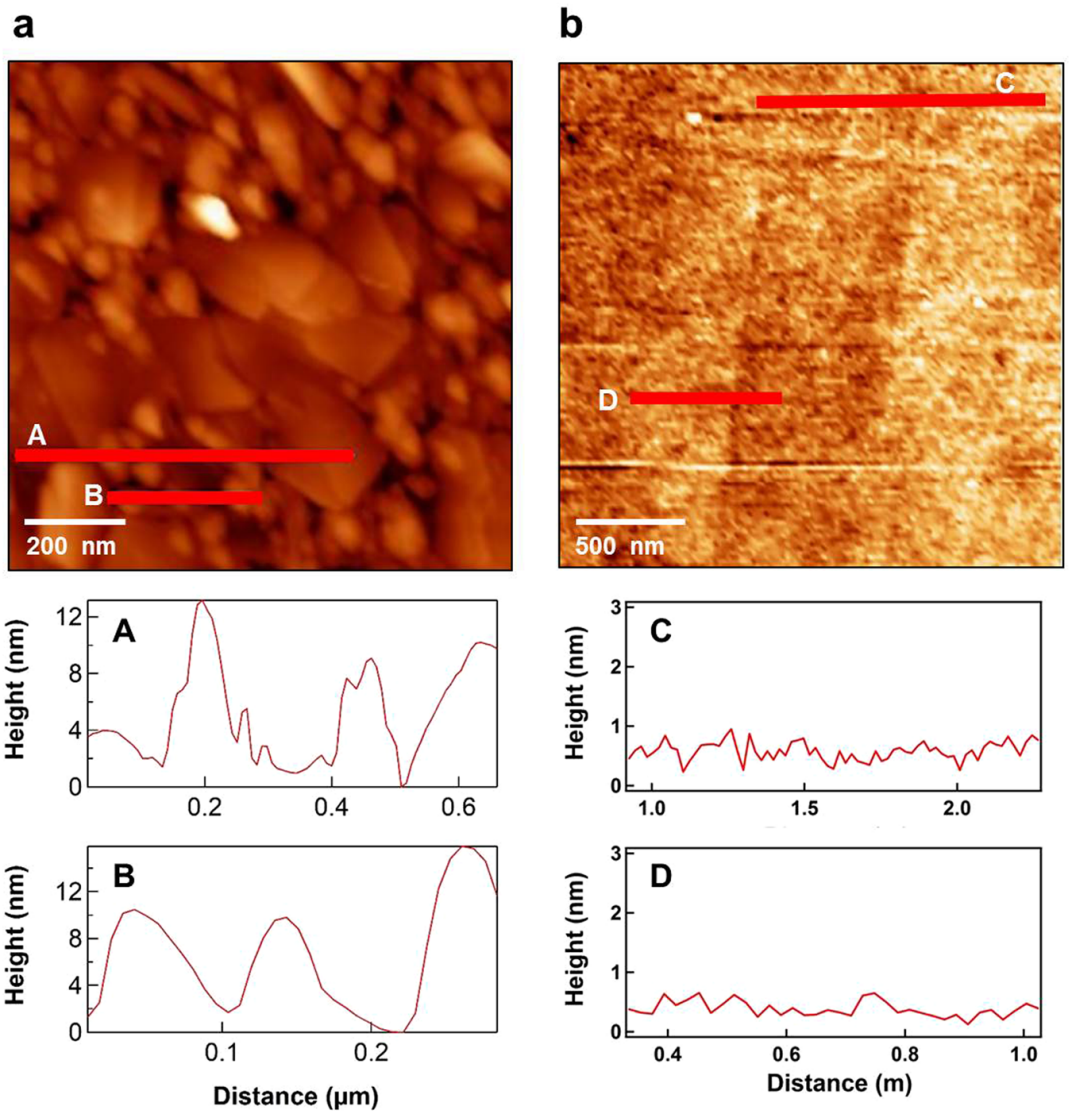
(**a**) AFM topography images and line profiles of a pristine MgO(001) before (**a**) and after 700 °C thermal annealing (**b**).

**Figure 3:**
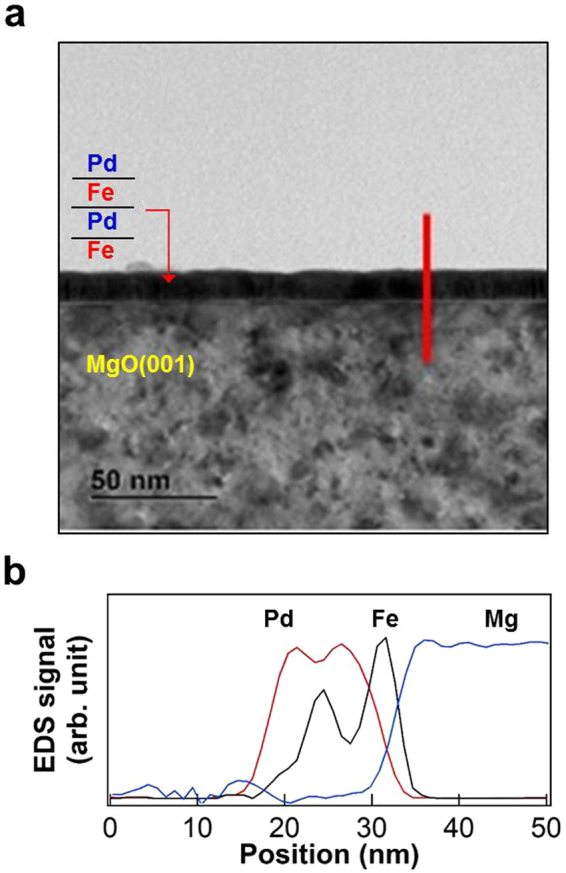
(**a**) Cross-sectional TEM image of a Pd/Fe/Pd/Fe thin film on MgO(001). (**b**) EDS element distribution line profiles of Pd (red), Fe (black) and Mg (blue) in the cross section, as indicated by the red line in (**a**).

Figure 2 compares the surface morphology of two MgO(001) substrates using AFM images^33^. One substrate was cleaned ultrasonically with 90% alcohol (Fig. 2a), whereas the other was annealed at 70 °C in vacuum better than 1 × 10^−7^ mbar (Fig. 2b). The pristine MgO(001) surface was completely covered by large structures up to hundreds of nanometer in scale, and small structures approximately tens of nanometer in scale, whereas the surface roughness fluctuated within 15 nm. As indicated by the line profiles in Fig. 2b, after thermal annealing, the MgO(001) surface had changed considerably: it became flat with a roughness of less than 1 nm. On this flat MgO(001) surface, prepared by 700 °C thermal annealing, uniform Fe/Pd multilayers were deposited to investigate Pd-mediated interlayer coupling. Figure 3 is the cross-sectional TEM image of a Pd/Fe/Pd/Fe thin film on MgO(001). The well-defined Pd/Fe multilayer structure was confirmed by the EDS line profiles plotted in Fig. 3b.

**Figure 4:**
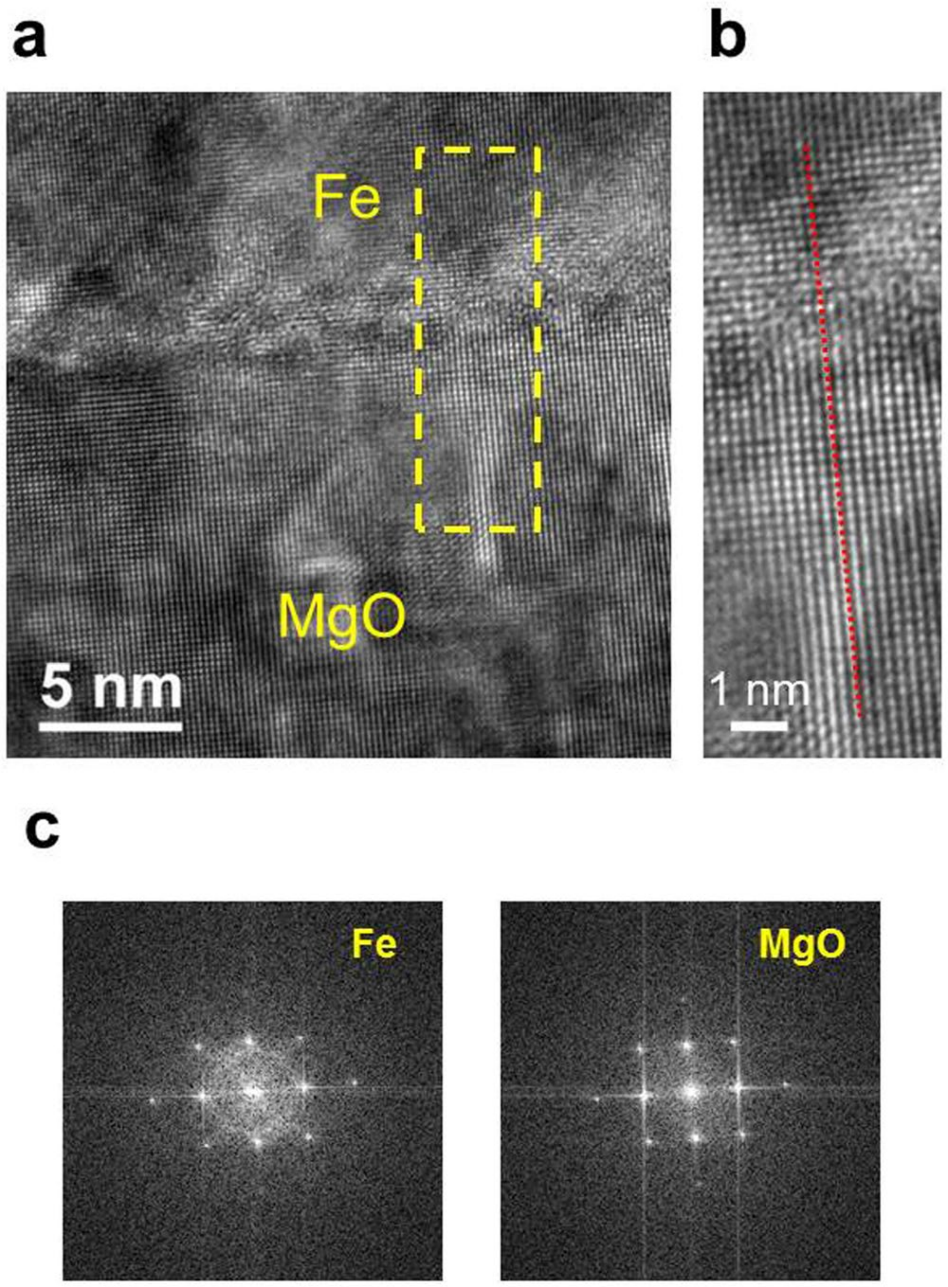
(**a**) A high-resolution TEM image near the Fe/MgO(001) interface region. (**b**) A magnified TEM image revealing the aligned crystalline structure of Fe(001) on MgO(001), as indicated by the red dashed line. (**c**) The fast Fourier transformation reveals the diffractograms of Fe (left) and MgO (right) under [1 0 0]Fe $\parallel$ [1 0 0]MgO zone.

As reported in previous studies, the lattice mismatch in Pd/Fe/MgO(001) was sufficiently small for coherent epitaxial growth^32^. Following rotation through 45°, the Fe(001) (lattice constant a_*Fe*_ = 2.87 Å) almost matched the MgO(001) (a_*MgO*_ = 4.21 Å), with evident mismatch of only approximately 4%^32^. Under this lattice matching condition, the bcc Fe 〈110〉 direction was aligned along the fcc MgO 〈100〉. For fcc Pd(001) (a_*Pd*_ = 3.89 Å) grown on bcc Fe(001), a small lattice mismatch of approximately 4% also ensured epitaxial growth^32^. Figure 4 depicts a high-resolution TEM image near the Fe/MgO(001) interface region. The magnified TEM image reveals the aligned crystalline structure of Fe(001) on MgO(001), as indicated by the red dashed line. Despite the sub-nanometer scale roughness of MgO, the atomic crystalline arrangement of Fe(001) was aligned along MgO(001). Figure 4c illustrates the Fourier transformation from two areas of Fe (left) and MgO (right), indicating the well-ordered cubic crystalline structure of Fe and MgO.

**Figure 5:**
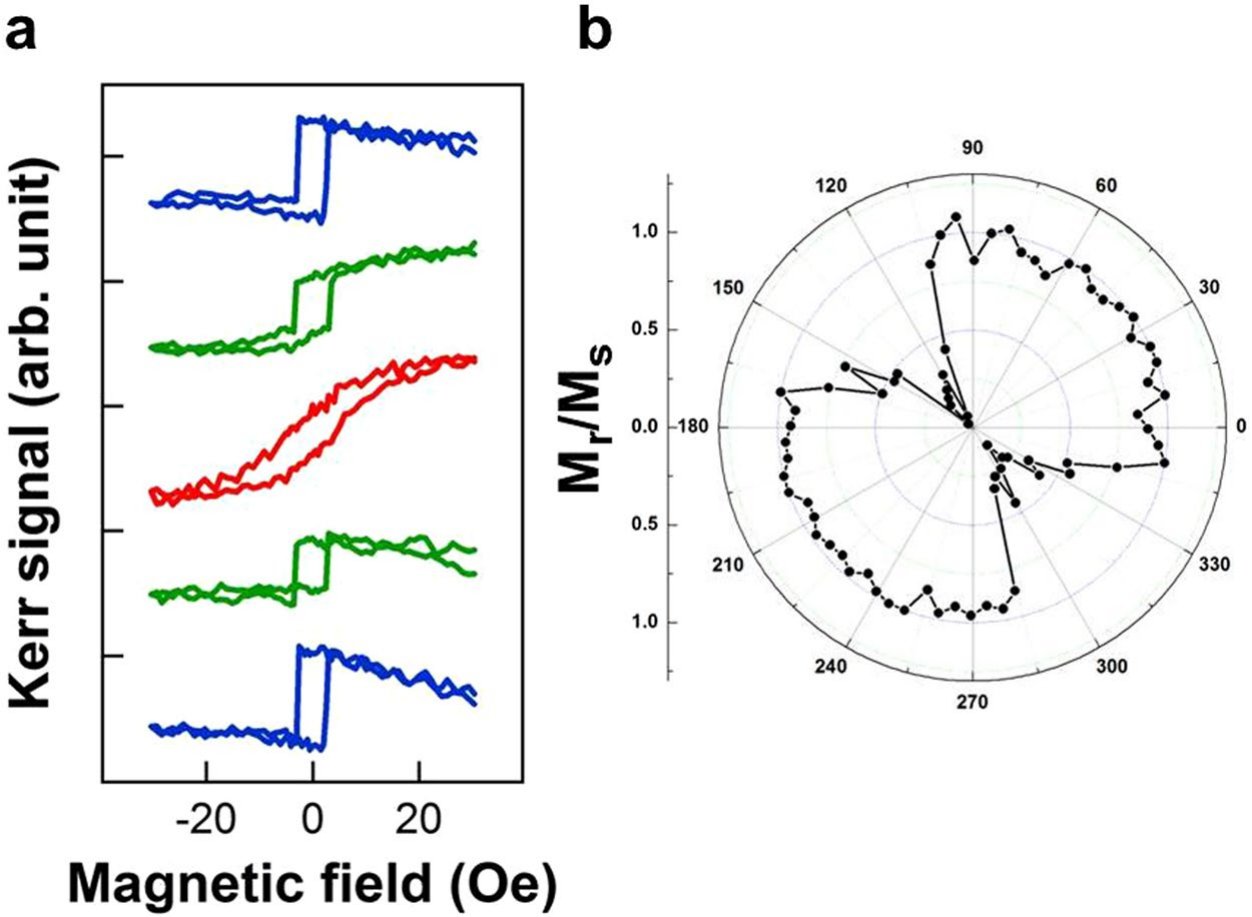
(**a**) MOKE hysteresis loops of 2-nm Pd/3-nm Fe measured at different azimuthal angle *φ*. (**b**) Polar illustration of the magnetic remanence (M_*r*_)/magnetic saturation (M_*s*_) ratio (i.e., squareness of hysteresis loop). Despite the 4-fold symmetry in the crystalline structure of Fe(001), the azimuthal angular dependent magnetic behavior indicates the dominance of a uniaxial magnetic anisotropy.

First, we explored the magnetic behavior of single-layer Fe on MgO(001). Figure 5a shows MOKE hysteresis loops of 2-nm Pd/3-nm Fe/MgO(001) measured at different azimuthal angle *φ*. At *φ* = 45° and 225°, the hysteresis loops were of a rectangular shape, the remanence (M_*r*_)/saturation (M_*s*_) ratio was nearly 100%, and the magnetization reversal curve was steep. At *φ* = 135°, the hysteresis loops became tilted, the M_*r*_/M_*s*_ ratio decreased to nearly 10%, and magnetization reversal occurred slowly and gradually. In Fig. 5b, in a polar illustration, the M_*r*_/M_*s*_ ratio, which represents the squareness of the hysteresis loop, is plotted as a function of the *φ*. The azimuthal angular dependence of the magnetic behavior indicated a bipolar symmetry, despite the quadropole symmetry in the crystalline structure of Fe(001). The bipolar symmetry was dominated by a uniaxial MAE, which may originate from the slight miscut of the substrate crystal orientation. This substrate morphology-induced uniaxial MAE is typically difficult to prevent and dominates collective magnetic behavior; this is particularly the case when the crystalline MAE is relatively small, as in the case of body centred cubic Fe.

### Magnetic coupling in Fe/Pd/Fe

**Figure 6:**
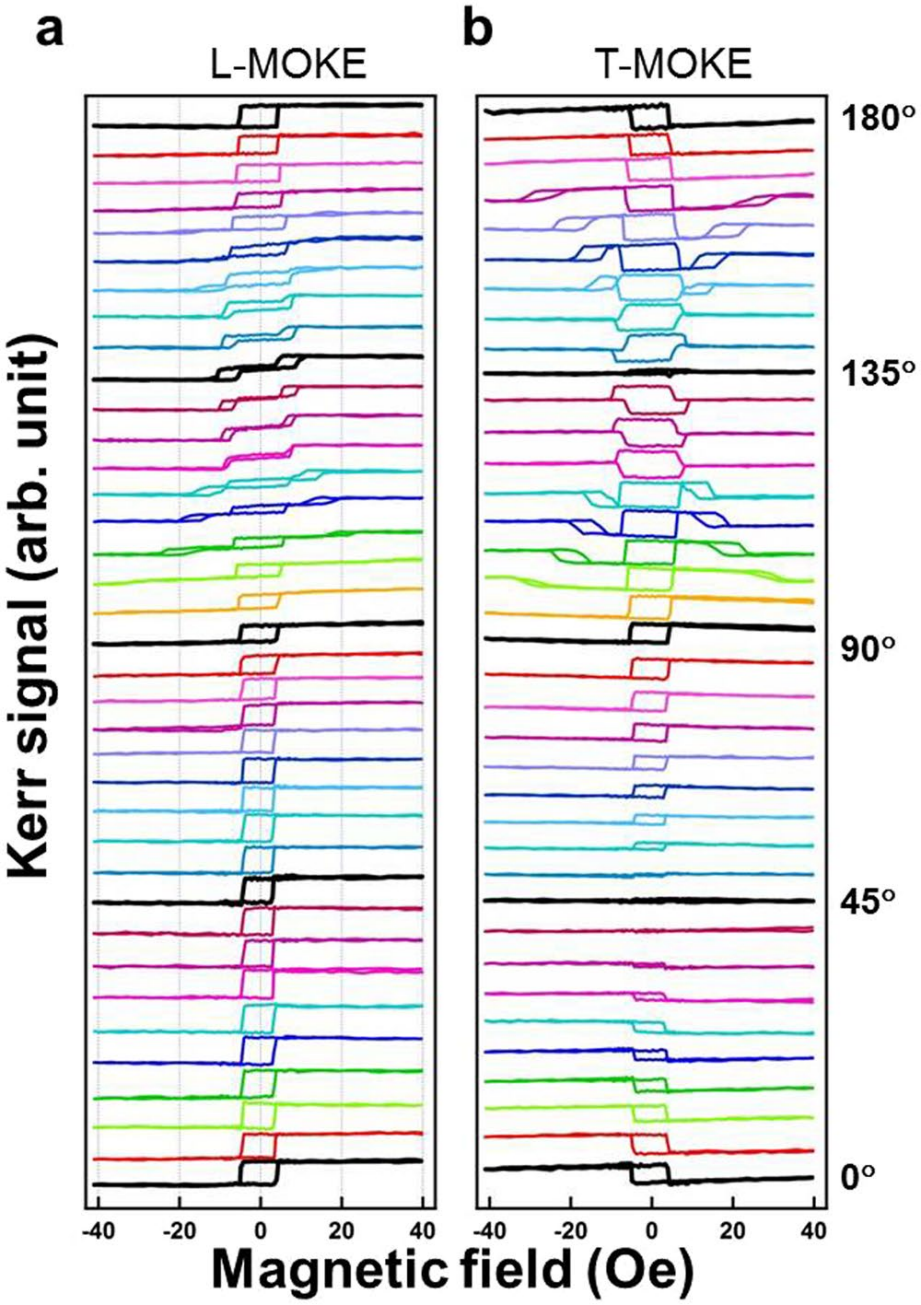
(**a**) Longitudinal and (**b**) transverse MOKE hysteresis loops of a 3-nm Pd/2-nm Fe/2-nm Pd/3-nm Fe thin film on MgO(001), measured with the azimuthal angle *φ* varied from 0° to 180°, at a step of 5°. Split minor loops were observed in the region of *φ* = 135° ± 30°.

On the basis of our understanding of the uniaxial MAE in the bottom layer Fe/MgO(001), we explored magnetic interlayer coupling in the Fe/Pd/Fe structure. Figure 6 depicts a series of (a) longitudinal MOKE (L-MOKE) hysteresis loops and (b) the corresponding transverse MOKE (T-MOKE) hysteresis loops for a sample of 3-nm Pd/2-nm Fe/2-nm Pd/3-nm Fe/MgO(001), measured at room temperature with a 0°–180° by a step of 5°. In the longitudinal MOKE measurement, square hysteresis loops persisted from *φ* = 0° to *φ* = 90°. With *φ* of approximately 105°, a square loop was evident in the center and two minor loops appeared in the positive and negative fields. When *φ* approached 135°, the height of center loop gradually reduced until it was invisible, and the two side loops moved toward the center. At *φ* = 135°, the longitudinal hysteresis loop evolved into a split double loop.

In the transverse MOKE measurement shown in Fig. 6(b), a square loop was observed at 0°. The height of the square hysteresis loops gradually decreased and became almost negligible with an increase of *φ* from 0° to 45°. When *φ* increased from 45° to 90°, a square hysteresis loop developed once more, this time with an inverse polarity. When *φ* was greater than 100°, we observed complex hysteresis behavior comprising a square center loop with two symmetric minor loops. When *φ* increased from 100° to 135°, the two minor loops gradually merged to the center loop. At *φ* = 135°, the Kerr intensity of the hysteresis loop decreased and was almost negligible compared with the others.

The MOKE measurement in Fig. 6 shows multi-symmetric magnetic behavior with the variation of *φ*. For example, the symmetrical evolutions in transverse MOKE in the regions of 45° ± Δ*φ* and 135° ± Δ*φ* implied the presence of a four-fold symmetry. However, in longitudinal MOKE, the distinct magnetic behavior at 0°–90° and 90°–180° implied that, not only a four-fold symmetry but also a two-fold symmetry of MAE must be considered^32^. Thus, a simple MAE model was proposed to explain this complex magnetic behavior.

**Figure 7:**
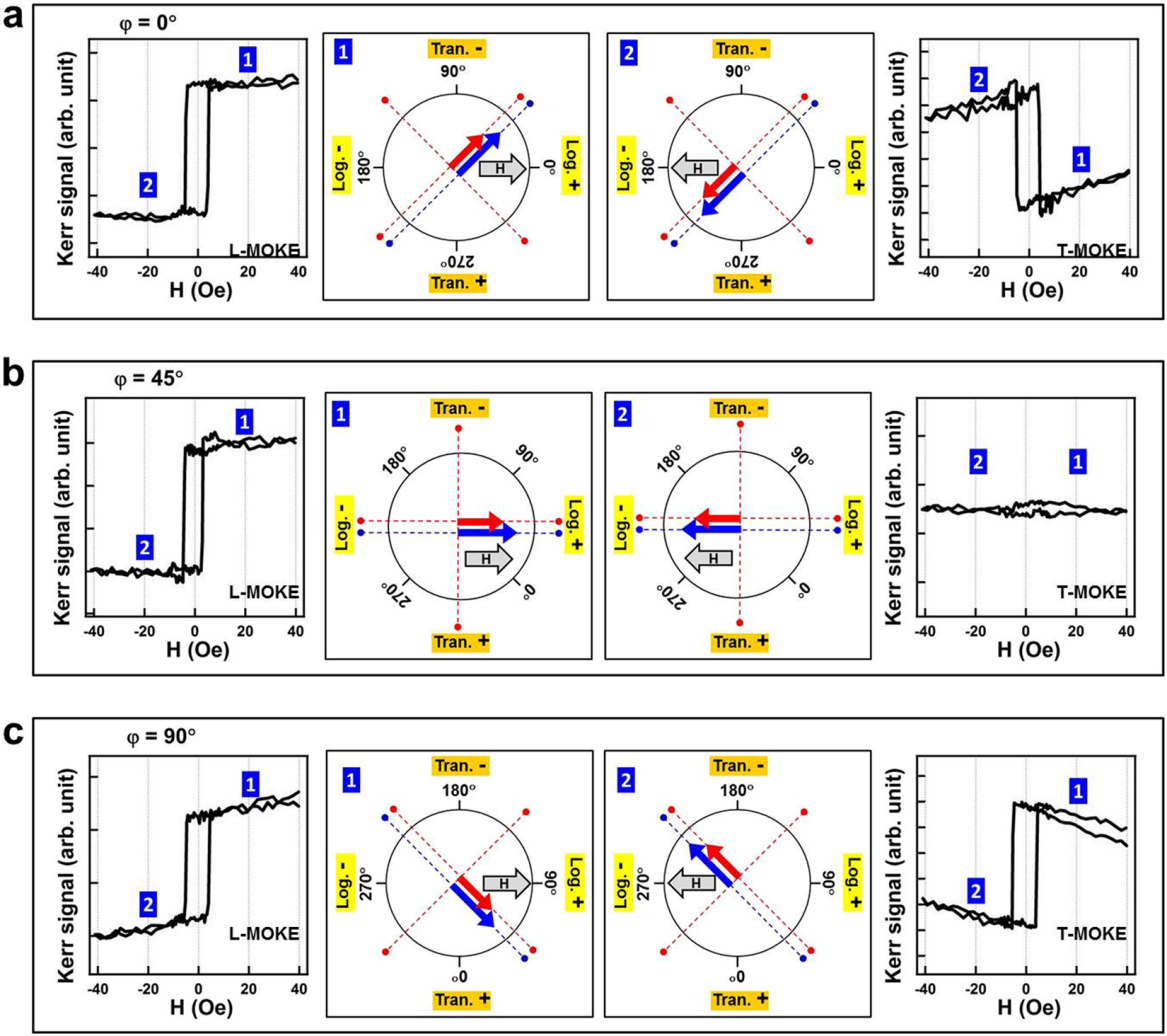
Illustration of the magnetization reversal mechanism for with the external magnetic field applied at *φ* = 0° (**a**), 45° (**b**) and 90° (**c**). The longitudinal and transverse MOKE hysteresis loops are shown in the left and right panels, respectively, enabling comparison with the proposed steps of magnetic moment-switching. The numbers denote the different magnetic states. In the schematic illustrations shown in the middle, red and blue arrows represent the magnetic moment of the top and bottom-Fe layer, respectively. Red and blue dashed lines indicate the 4- and 2-fold magnetic easy directions for the top- and bottom-Fe layers, respectively. The gray arrow indicates the direction of the external magnetic field; longer the gray arrow, larger was the magnetic field applied.

**Figure 8:**
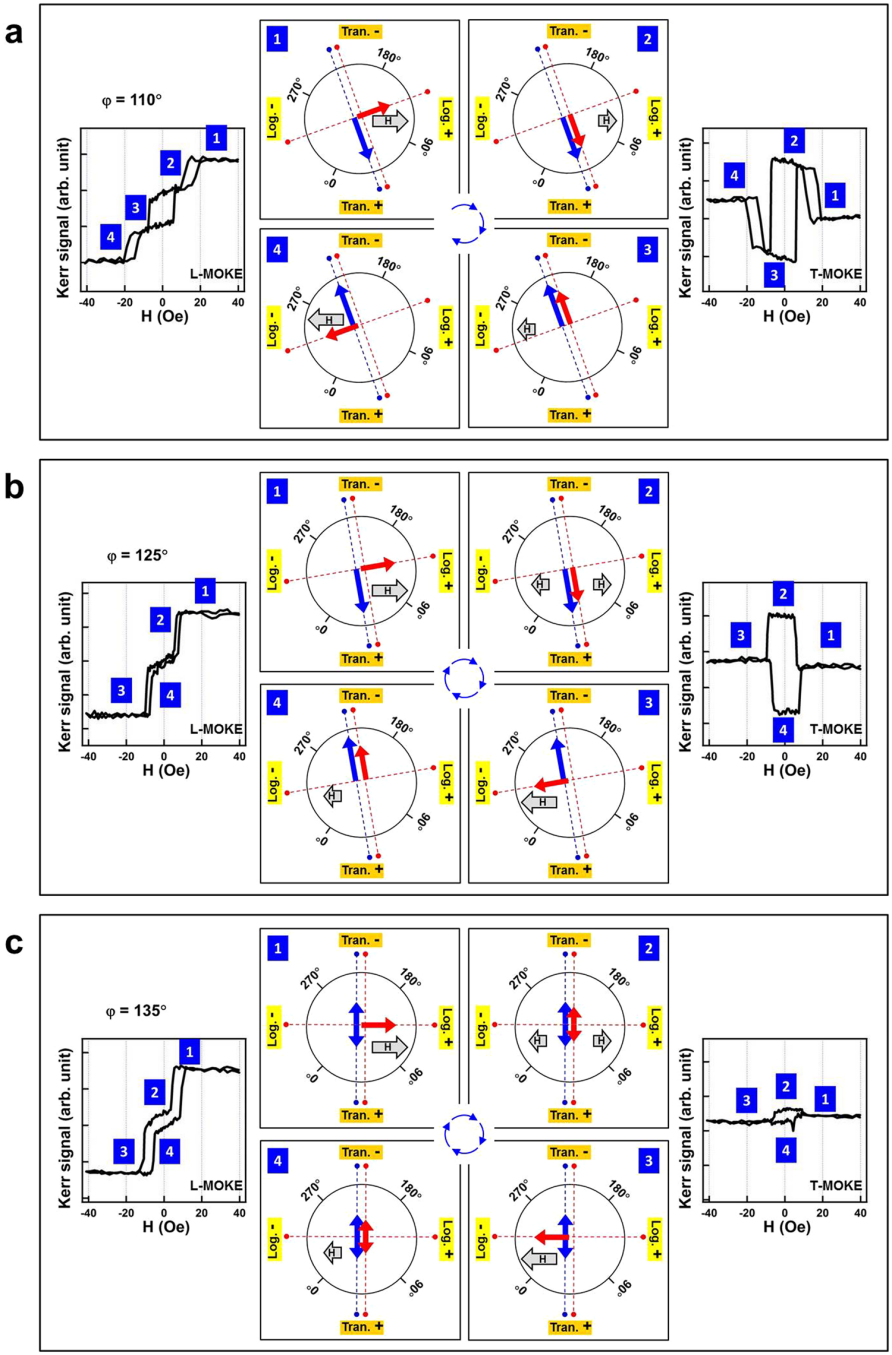
Illustration of the magnetization reversal mechanism with the external magnetic field applied at *φ* = 110° (**a**), 120° (**b**) and 135° (**c**). The longitudinal and transverse MOKE hysteresis loops are shown in the left and right panels, respectively, enabling comparison with the proposed steps of magnetic moment switching. The numbers indicate the corresponding order of the different magnetic states. In the schematic illustrations shown in the middle, red and blue arrows represent the magnetic moment of the top- and bottom-Fe layers, respectively. Red and blue dashed lines indicate the 4- and 2-fold magnetic easy directions for the the top- and bottom-Fe layers, respectively. The gray arrows indicate the direction of the external magnetic field; the longer the gray arrow, larger was the magnetic field applied.

In the present double Fe layer system, the bottom-Fe layer was affected by the miscut of the substrate and therefore a uniaxial (2-fold) MAE dominated the magnetic behavior in a manner similar to that shown in Fig. 5. The top-Fe layer was less affected by the substrate and therefore its intrinsic crystalline (four-fold) MAE dominated. The azimuthal angle *ϕ* = 0° was parallel to the 〈100〉 direction of the MgO(001) substrate. Because the deposited Fe(001) film rotated 45° to match the lattice of MgO(001), *ϕ* = 45° is parallel to the 〈110〉 direction of the MgO(001) substrate, that is the 〈100〉 direction of the Fe(001) film as well. Thus the proposed 4-fold easy directions of top-Fe along *ϕ* = 45° and 135°, as shown in Figs 7–8, are consistent with the conventional easy axis of fcc-Fe: the 〈100〉 directions^29,32^. Considering the hypothesis regarding the combination of a 2-fold MAE in bottom-Fe and a 4-fold MAE in the top-Fe, the complex MOKE hysteresis loops can be clearly understood (Figs 7 and 8).

Figures 7 and 8 provide several examples of *φ* = 0°, 45°, 90°, 110°, 125°, and 135°, where the detailed magnetization reversal processes are explained through comparison with the experimental L- and T-MOKE data. The longitudinal and transverse MOKE hysteresis loops are shown in the left and right panels of these figures, respectively, thus providing a comparison with the proposed magnetic moment-switching steps. The numbers indicate the corresponding order of the different magnetic states. In the schematic in the center, the gray arrows represent the direction of the external magnetic field; longer the gray arrow, larger was the magnetic field applied. The red and blue arrows represent the magnetic moment of the top- and bottom-Fe layers, respectively. Red and blue dashed lines indicate the 4- and 2-fold magnetic easy directions for the top- and bottom-Fe layers, respectively. For the bottom-Fe layer, the magnetic easy axis lay along the 45°-axial orientation. For the top-Fe layer, the magnetic easy axis lay along the 45°- and 135°-axial orientations. Because the two Fe layers were ferromagnetically coupled and the 45°-axial orientation was the magnetic easy axis for both Fe layers, the magnetic moments of individual layers were bound together; when the external magnetic field was applied at a *φ* close to 45° (i.e., within 45° ± 45°, as illustrated in Fig. 7), square hysteresis loops were observed and measured. As shown in Fig. 8, when the magnetic field was large and applied at an *φ* almost perpendicular to the easy axis of 45° (i.e., *φ* within 135° ± 45°), the top-Fe moment could be aligned by the field to the other easy axis of 135° and simultaneously decoupled from the bottom-Fe layer.

We observed the nearly same coercive field (∼5 Oe) and rectangular shape in the L-MOKE loops of Fig. 7a and c. These features pointed out the symmetric behavior at *ϕ* = 0° and *ϕ* = 90°. The difference between the background base-lines, could originate from the imperfect crystalline symmetry in the sample or the optical background signal in MOKE setup. Unlike the measurement of absolute magnetization moment by SQUID or VSM, MOKE measured the surface-sensitive magnetic hysteresis loops, which was usually coupled with the Faraday effect from optical components and intensity drift of light source. As the directions of *ϕ* = 0° and *ϕ* = 90° were neither along the easy axes, a slanted and unsaturated background curve was expected. However because the maximum field used in Fig. 7 was far below the saturation field, the expected slated curve was indistinct, within the background signal of MOKE.

As illustrated in Fig. 8a, when the magnetic field was applied along *φ* = 110°, a triple-loop was observed in both L- and T-MOKE. In State 1, a large positive magnetic field could sustain the top-Fe layer along the easy direction of 135°, whereas the bottom-Fe layer remained in its uniaxial easy direction of 45°, leading to decoupling of the two Fe layers. In State 2, the positive magnetic field was reduced to a smaller magnitude that was insufficient small to sustain the top-Fe layer along 135°; thus, the top-Fe layer flipped to 45°, the same direction as the bottom-Fe layer, owing to ferromagnetic interlayer coupling. In State 3, the magnetic field changed to a negative direction; a small negative field was sufficient to switch the bound Fe layers from 45° to 225°, which remained along their common easy axis. In State 4, the negative magnetic field was increased to a larger magnitude, sufficient to align the top-Fe layer to 315°. This four-step magnetization process can be qualitatively confirmed from the Kerr signal variation measured in L- and T-MOKE curves.

When the magnetic field was applied along a *φ* angle close to the 135° easy axis of the top-Fe layer, stronger Zeeman energy reduced the critical field in the State 3-to-4 transition (Fig. 8a). Thus, the State-2-to-3 and State-3-to-4 transitions (Fig. 8a) occurred almost simultaneously when *φ* was rotated to 125°; that is the State-2-to-3 transition depicted in Fig. 8b. Accordingly the triple-loop at 110° (Fig. 8a) merged to form a double loop at 125° (Fig. 8b). As shown in Fig. 8(c), when the magnetic field was rotated to 135° and was perpendicular to the easy axis of the bottom-Fe layer, the bottom-Fe layer exhibited no preference for either of the two easy directions. When the strength of the magnetic field was insufficient to decouple the two Fe layers (i.e., the field was close to zero), the equal distribution of the bottom-Fe layer in both easy directions pinned the top-Fe layer along the positive and negative transverse directions; thus, we obtained a T-MOKE loop substantially smaller than 110°–130°.

Ferromagnetic-metal/normal-metal(NM) multilayers usually present ferromagnetic or antiferromagnetic interlayer coupling with appropriate thickness of the NM layer^22,23^. Until now only few studies have investigated Pd-mediated RKKY interlayer exchange coupling. In ultrathin Fe(001)/Pd(001)/Fe(001)/Ag(001) structures, Celinski *et al*. reported the ferromagnetic interlayer coupling at Pd thickness less than 12 MLs (2.5 nm). When the thickness was of 13–16 MLs (2.7–3.4 nm), it displayed weak long-range antiferromagnetic coupling^24,29,30^. However, in epitaxial Fe(001)/Pd superlattices on MgO(001), Childress *et al*. concluded that no evidence for antiferromagnetic coupling between Fe layers through Pd interlayers could be found in Pd with a thickness of 1–5 nm^32^. Our measurement results of [Pd/Fe]_2_/MgO(001) are consistent with Childress *et al*’s report: no observable AFM coupling with Pd thickness between 1–5 nm. This means that the epitaxial Fe/Pd/Fe trilayer on Mg(001) always revealed ferromagnetic coupling, which monotonically decayed with the increase of Pd thickness.

### Effect of hydrogenation on long-range-coupled Fe/Pd/Fe

**Figure 9:**
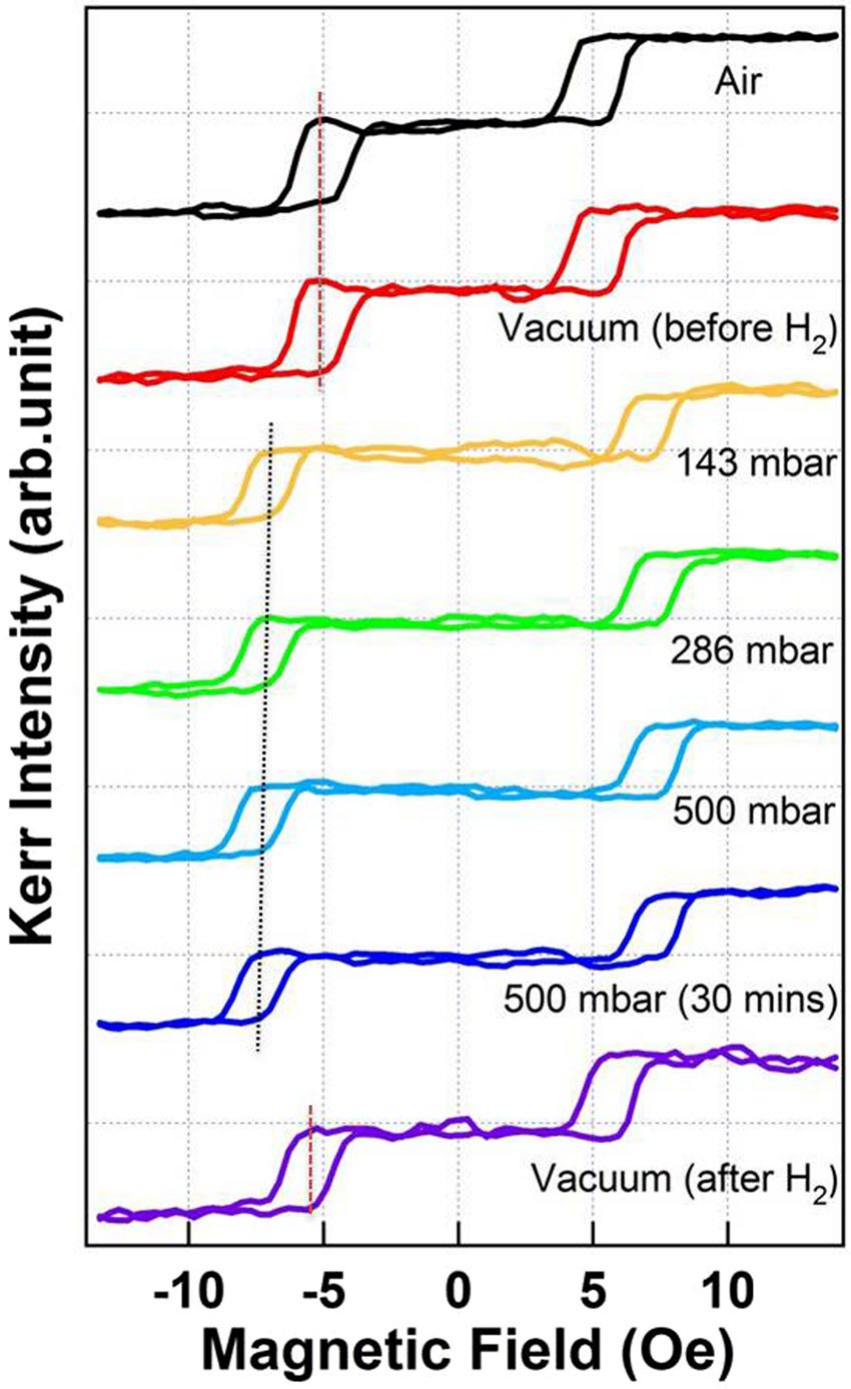
Longitudinal MOKE hysteresis loops of 3-nm Pd/2-nm Fe/3-nm Pd/4-nm Fe measured under varying H_2_ gas pressure, with the magnetic field perpendicular to the easy axis of the bottom-Fe layer,. The double minor loops originated from the 90° rotation of the top-Fe layer. The dashed lines indicate the center of the double loops.

**Figure 10:**
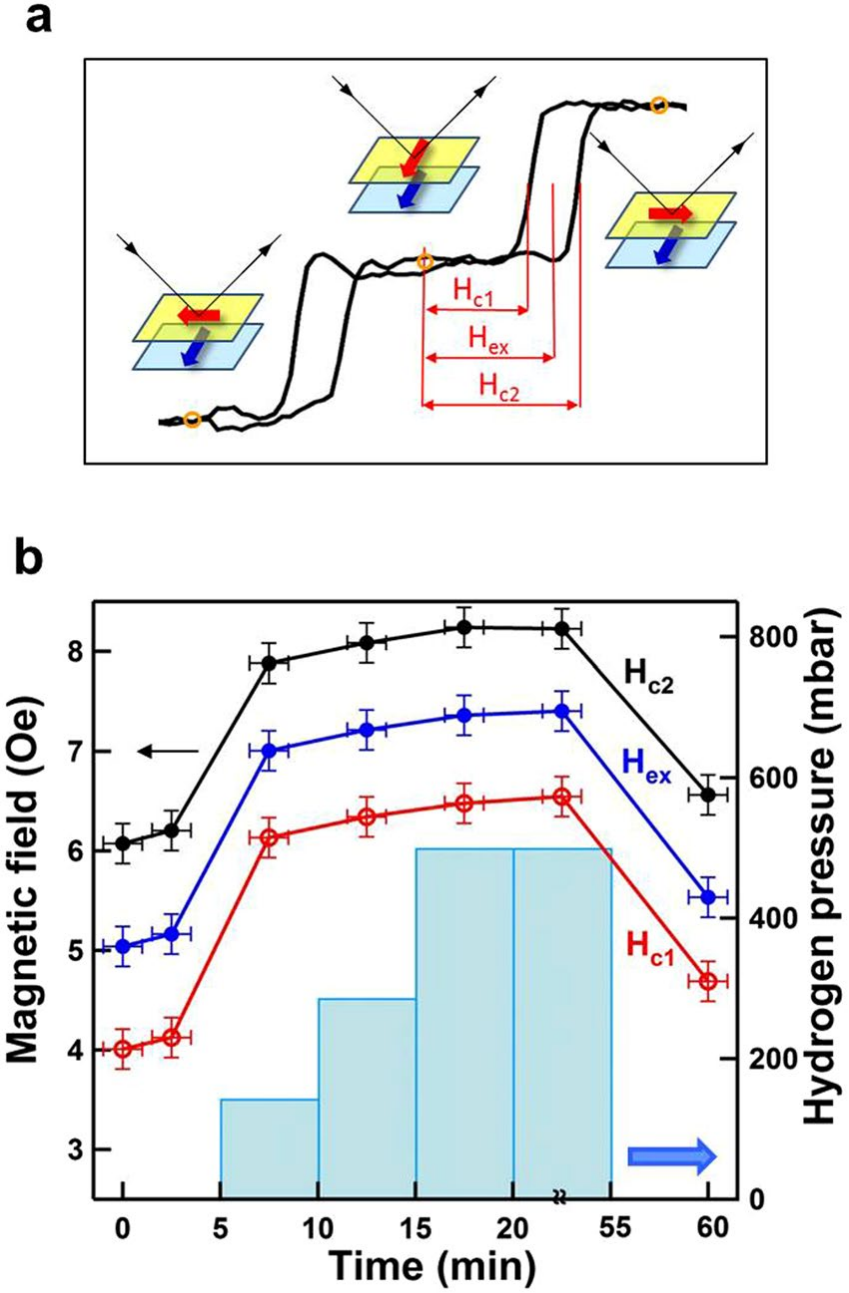
(**a**) Schematic of the three different magnetization ordering of Fe/Pd/Fe in the double loop measured in Fig. 9. H_*c*1_, H_*c*2_ and H_*ex*_ were defined as first coercivity, second coercivity and center of the minor loop, respectively. (**b**) Left: magnetic field of H_*c*1_, H_*c*2_ and H_*ex*_ deduced from Fig. 8. Right: corresponding H_2_ gas pressure in the measurement environment.

To investigate the effect of hydrogenation on long-range coupling in Fe/Pd/Fe, we focused on the longitudinal double hysteresis loop, which lies at *φ* = approximately 130° (Fig. 6). Figure 9 shows the double hysteresis loops of 3-nm Pd/2-nm Fe/3-nm Pd/4-nm Fe, which were measured, under varying H_2_ gas pressures, with the magnetic field perpendicular to the easy axis of the bottom-Fe layer. The dashed lines indicate the center of the minor loops. The measurements in air and vacuum revealed almost identical hysteresis loops, suggesting that the coercivity field of the double loop was invariant. When the sample was exposed to 143–500 mbar H_2_, the minor loops shifted toward a larger magnetic field. When H_2_ gas was pumped out of the measurement chamber, recovering the vacuum, the minor loops returned to the pristine coercivity field. This demonstrates the reversibility of the hydrogenation-induced shift of minor loops. For quantitative analysis, H_*c*1_, H_*c*2_, and H_*ex*_ were defined as the first coercivity, the second coercivity, and the center of minor loop, respectively, as illustrated in Fig. 10a. Figure 10b displays the magnetic field of H_*c*1_, H_*c*2_, and H_*ex*_ (left axis) with the corresponding H_2_ gas pressure in the measurement environment (right axis). On exposure to 143 mbar H_2_ gas, the H_*c*1_, H_*c*2_ and H_*ex*_ simultaneously increased by 2.5 ± 0.2 Oe. Further increase in H_2_ gas pressure to 500 mbar shifted the H_*c*1_, H_*c*2_, and H_*ex*_ upward by 0.5 ± 0.2 Oe. Notably, although hydrogenation moved the minor loops toward a larger magnetic field, the width of the minor loop (i.e., H_*c*2_ − H_*c*1_) was almost invariant, at least within the margin of error.

In Fig. 9 the hydrogen-induced changes in MOKE hysteresis loops were plotted with the various hydrogen gas pressures. In the bottom panel, the hysteresis loop measured after pumping hydrogen gas out from the chamber (i.e. the recovery of a vacuum) was exhibited for the demonstration of the reversibility. We can clearly observe that the split double loops moved toward small filed direction, almost recovering the initial MOKE loop in the pristine vacuum condition. The quantitative analysis in Fig. 10 also shows that the hydrogen effect on H_*ex*_ was reversible. When the sample environment recovered a vacuum, the hydrogen desorption rate was much higher than the absorption rate. Most of hydrogen leaved the sample and the magnetic property recovered the pristine condition. The respond time constants for the hydrogen absorption under H_2_ gas environment and the hydrogen desorption in a vacuum ranged few minutes, which could be reduced to few seconds by making the multilayers into a nanostructure^15^.

## Discussion

The uniaxial MAE of the bottom-Fe on MgO(001) was attributed to the miscut of the substrate. In previous reports, the miscut of the substrate has been demonstrated to cause a uniaxial MAE in the deposited thin film^34^. The possible physical origins are the atomic step-induced interfacial MAE, the elongated terrace-induced shape MAE and etc. In our experiment, this observed uniaxial MAE always existed in various samples of Fe/MgO(001). This is because the crystalline MAE of bcc-Fe is relatively small and thus easy to modulate, by the surface-miscut for example. Because of the random polishing-direction of the MgO(001) substrates, the direction of the observed easy axis was not fixed, but mostly located at *ϕ* = 0° or *ϕ* = 45°. Thus we have to choose suitable uniaxial direction (along *ϕ* = 45°) to reproduce the similar experimental results as Fig. 9. In future applications, not only the miscut of MgO(001), but also the oblique deposition of Fe can be the promising methods to reproduce the uniaxial MAE along certain direction^35,36^.

The complex magnetic hysteresis loops (Fig. 7) originated from the coexistence of a uniaxial MAE and a cubic MAE. As shown in Fig. 5, the uniaxial MAE originated from the bottom-Fe layer, potentially caused by the possible slight tilting of MgO(001) surface orientation. Because of the relatively small cubic MAE of bcc Fe, this uniaxial MAE dominated the magnetic behavior of the bottom layer, which exhibited 2-fold symmetric magnetic properties (Fig. 5b). The effect of substrate morphology on the subsequently deposited Pd-mediate layer and the top-Fe layer reduced gradually. Consequently, the intrinsic cubic MAE dominated the top-Fe layer; simultaneously, the top- and bottom-Fe layers were ferromagnetically coupled through the Pd-mediate layer. The long-range interlayer coupling energy can be estimated simply by E_*ex*_ ≈ H_*ex*_⋅M_*top*−*Fe*_ ≈ 2.5 × 10^−3^ erg/cm^2^. All the magnetic energy, including uniaxial MAE, cubic MAE, and interlayer coupling, were comparable. Consequently, the interplay between these energy forms induced the complex magnetization reversal behavior in the azimuthal-dependent hysteresis loops.

As shown in Fig. 9, although hydrogenation shifted the minor loops toward a larger magnetic field, the width of the minor loop remained invariant. Furthermore, as illustrated in Figs 8 and 10a, the double minor loops originated from the 90° rotation of the top-Fe layer. The H_*ex*_ correlated with the exchange coupling between the two Fe layers, while the width of the minor loop correlated with the local trapping strength (potential depth) of the top-Fe in the quadropole MAE. As shown in Fig. 10, H_*ex*_ increased from 5 Oe in vacuum to 7 Oe under 143–500 mbar H_2_. As estimated by E_*ex*_ ≈ H_*ex*_Â·M*top* − *Fe*, the magnetic interlayer coupling energy E_*ex*_ between the two Fe films increased from 2.5 × 10^−3^ to 3.5 × 10^−3^ erg/cm^2^. Although the magnetic interlayer coupling was weak, the hydrogen-induced enhancement was substantial, reaching as high as 40%. The observation of enhanced E_*ex*_ indicated that the hydrogenation of Pd-covered Fe/Pd/Fe strengthened long-range-coupling between the two Fe layers. Moreover, the stable width of the minor loops indicated the invariant cubic MAE of the top-Fe layer. This conclusion is straightforward and reasonable given that the Pd-H hydride formation was more energetically favorable than was the Fe-H hydride. Studies have reported that 500 mbar H_2_ can provide a composition of about 0.7 hydrogen/metallic atom in pure Pd and that of less than 0.01 hydrogen/metallic atom in Fe_0.074_Pd_0.926_^37^. Because 7.4% Fe content in the Fe-Pd alloy can considerably reduce the composition of absorbed hydrogen, the hydride formation in pure Fe is assumed to be negligible.

**Figure 11:**
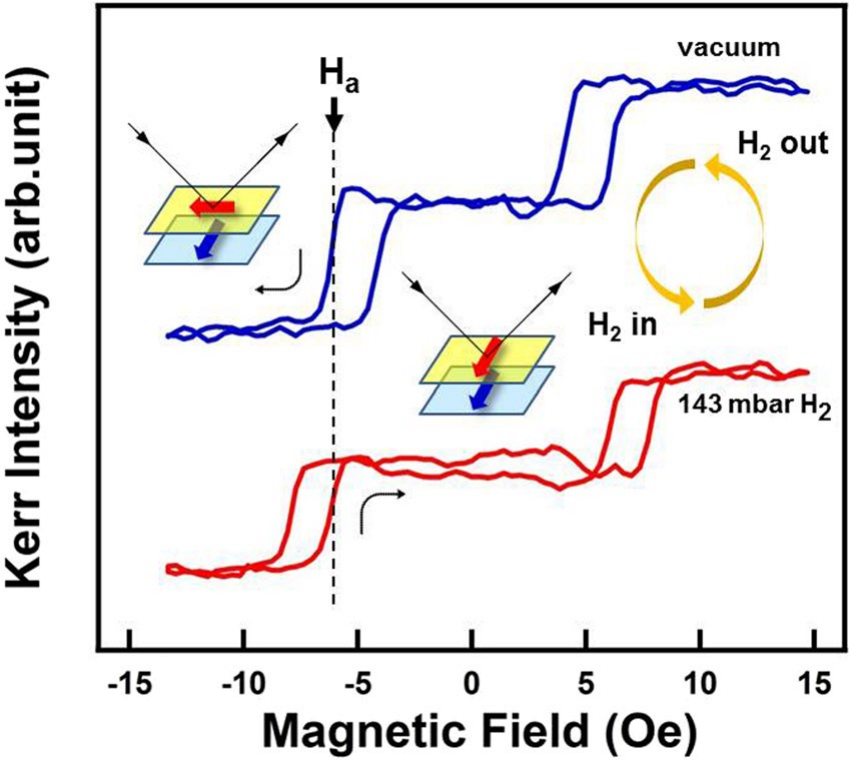
Reversible 90° rotation of the magnetic moment in the top Fe layer through hydrogen charging. Following H_2_ gas absorbtion or desorption, the magnetization minor hysteresis loop shifted toward a larger or smaller coercivity field. When a suitable constant field H_*a*_ (as indicated by the dashed line) was applied, the hydrogen charging or discharging reverses the top-Fe layer by 90°.

Because of the reversible hydrogenation-induced change in interlayer coupling in the present Fe/Pd/Fe system, we can directly control the magnetic ordering of the two Fe layers by hydrogen exposure. As illustrated in Fig. 11, if a constant biasing magnetic field H_*a*_ ≈ 6 Oe is applied to the Fe/Pd/Fe sample, the hydrogen that is charged or discharged will shift the minor loop leftward or rightward, thus causing a reversible 90° rotation of the top-Fe layer. This effect can be readily applied for magnetoresistance, such as that in a GMR device, for hydrogen sensing.

## Conclusion

[Pd/Fe]_2_ multilayers were deposited on a flat MgO(001) substrate to investigate the effect of hydrogen on interlayer coupling. The characterization of a crystalline structure using high-resolution TEM confirmed the coherent growth of Pd/Fe on MgO(001). For single-layer Fe on Mg(001), a uniaxial MAE was observed, which is attributable to the possible miscut of the substrate. In the Fe/Pd/Fe interlayer coupled system, complex magnetic hysteresis behavior, including single, double, and triple loops, was measured as a function of the *φ* in a longitudinal and transverse direction. The bottom-Fe layer was affected by the miscut of the substrate and therefore a uniaxial (2-fold) MAE dominated the magnetic behavior. The top-Fe layer was not affected by the substrate considerably and its intrinsic crystalline (four-fold) MAE was more likely to dominate. Following the hypothesized combination of 2- and 4-fold MAE in the bottom- and top-Fe layers, respectively, the complex MOKE hysteresis loops can be clearly explained. By selecting a suitable Pd thickness and applying the magnetic field perpendicular to the easy axis of the bottom-Fe layer, two well-split hysteresis loops with almost zero Kerr remanence were measured. These split double loops originated from the 90° rotation of the top-Fe moment. Through exposure to a hydrogen gas atmosphere, separation of the two minor loops increased owing to the Pd-hydride formed in the space layer. The reversibility of this hydrogenation effect was then demonstrated. The increased switching field indicated that Pd-hydride formation enhanced ferromagnetic interlayer coupling between the top- and bottom-Fe layers. Thus, we can conclude that when a suitable constant magnetic field is applied, the top-Fe moment will undergo a reversible 90° rotation following hydrogen exposure. These results suggest that the Pd space layer, which mediates the magnetic interlayer coupling, is sensitive to the hydrogen atmosphere; the [Fe/Pd]_*n*_ multilayer system can therefore function as a GMR-type sensor suitable for hydrogen sensing.

## Methods

[Pd/Fe]_*n*_ multilayer thin films were deposited on MgO(001) substrates using e-beam-heated evaporators. The surface morphology of the sample was investigated using a Bruker Multimode 8 atomic force microscope (AFM). The characterizations of transmission electron microscopy (TEM) were conducted by using TECNAI G2 F20 TEM equipped with Oxford X-MAX80 X-ray Energy Dispersive Spectrum (EDS) detector. For both longitudinal and transverse geometries, the magnetic properties of the Co-Pd films were investigated at room temperature using a magneto-optical Kerr effect (MOKE). MOKE measurements were conducted in a small vacuum chamber with windows. To investigate the effect of hydrogen on the magneto-optical and intrinsic magnetic properties of the Co-Pd alloy films, the MOKE chamber was pumped to a vacuum of 5 × 10^−3^ mbar or filled with H_2_ gas at various pressures during measurement.
